# Brain–Immune Interaction Accompanying Odor-Evoked Autobiographic Memory

**DOI:** 10.1371/journal.pone.0072523

**Published:** 2013-08-20

**Authors:** Masahiro Matsunaga, Yu Bai, Kaori Yamakawa, Asako Toyama, Mitsuyoshi Kashiwagi, Kazuyuki Fukuda, Akiko Oshida, Kazue Sanada, Seisuke Fukuyama, Jun Shinoda, Jitsuhiro Yamada, Norihiro Sadato, Hideki Ohira

**Affiliations:** 1 Department of Health and Psychosocial Medicine, Aichi Medical University School of Medicine, Aichi, Japan; 2 Department of Psychology, Graduate School of Environmental Studies, Nagoya University, Aichi, Japan; 3 Kao Corporation, Kansei Science Research Laboratories, Tokyo, Japan; 4 Kao Corporation, Perfumery Development Research Laboratories, Tokyo, Japan; 5 Chubu Medical Center for Prolonged Traumatic Brain Dysfunction, Kizawa Memorial Hospital, Gifu, Japan; 6 Division of Cerebral Integration, Department of Cerebral Research, National Institute for Physiological Sciences, Aichi, Japan; Hospital General Dr. Manuel Gea González, Mexico

## Abstract

The phenomenon in which a certain smell evokes a specific memory is known as the Proust phenomenon. Odor-evoked autobiographic memories are more emotional than those elicited by other sensory stimuli. The results of our previous study indicated that odor-evoked autobiographic memory accompanied by positive emotions has remarkable effects on various psychological and physiological activities, including the secretion of cytokines, which are immune-signaling molecules that modulate systemic inflammation. In this study, we aimed to clarify the neural substrates associated with the interaction between odor-evoked autobiographic memory and peripheral circulating cytokines. We recruited healthy male and female volunteers and investigated the association between brain responses and the concentration of several cytokines in the plasma by using positron emission tomography (PET) recordings when an autographic memory was evoked in participants by asking them to smell an odor that was nostalgic to them. Participants experienced positive emotions and autobiographic memories when nostalgic odors were presented to them. The levels of peripheral proinflammatory cytokines, such as the tumor necrosis factor-α (TNF-α) and interferon-γ (IFN-γ), were significantly reduced after experiencing odor-evoked autobiographic memory. Subtraction analysis of PET images indicated that the medial orbitofrontal cortex (mOFC) and precuneus/posterior cingulate cortex (PCC) were significantly activated during experiences of odor-evoked autobiographic memory. Furthermore, a correlation analysis indicated that activities of the mOFC and precuneus/PCC were negatively correlated with IFN-γ concentration. These results indicate that the neural networks including the precuneus/PCC and mOFC might regulate the secretion of peripheral proinflammatory cytokines during the experience of odor-evoked autobiographic memories accompanied with positive emotions.

## Introduction

The phenomenon in which a certain smell evokes a specific memory—for example, the smell of a madeleine biscuit dipped in linden tea triggering intense joy and the memory of childhood [1]—is known as the “Proust phenomenon.” Previous studies have characterized odor-evoked autobiographic memories as being more emotional than those elicited by other sensory stimuli [2–5]. The mechanism underlying the observation that odor-evoked autobiographic memories are quite emotional might be the direct neural communication between the olfactory system and the amygdala–hippocampal complex of the limbic system, which is directly involved in basic-level emotion and memory processing [6,7]. In fact, a previous neuroimaging study has indicated that the amygdala is strongly activated when autobiographic memories are evoked by olfactory cues than by other sensory cues, such as visual ones [5].

Recent studies have shown that emotional experiences can induce various psychological and peripheral physiological responses involving the autonomic nervous, endocrine, and immune systems [8–14]. Thus, experiencing odor-evoked autobiographic memories may also induce several psychological and physiological responses. We have recently shown that odor-evoked autobiographic memories accompanied by positive emotions have an inhibitory effect on the secretion of peripheral cytokines, which are immune-signaling molecules that modulate systemic inflammation [10]. After infection with some pathogens, systemic inflammation is usually induced via the active secretion (from immune cells) of proinflammatory cytokines, such as the tumor necrosis factor-α (TNF-α) and interferon-γ (IFN-γ), which can induce depressive symptoms [15]. Recent studies in psychoneuroimmunology have shown that emotional experiences can modulate the secretion of proinflammatory cytokines from immune cells, and that psychological stressors, such as anxiety, can induce the active secretion of proinflammatory cytokines [14]. In contrast, other studies have shown that pleasurable experiences, such as the evocation of happy feelings, play a role in suppressing the secretion of proinflammatory cytokines and have various beneficial effects on health and well-being [9]. Therefore, pleasurable experiences induced by odor-evoked autobiographic memory might also have remarkable beneficial effects on human health and well-being via the inhibition of systemic inflammation.

The neural underpinnings of the association between odor-evoked autobiographic memory accompanied by positive emotions and peripheral circulating cytokines remain obscure. Our previous neuroimaging studies, which focused on the brain–immune interaction, have shown that the prefrontal brain regions, such as the ventromedial prefrontal cortex (vmPFC) and the orbitofrontal cortex (OFC), regulate peripheral immune activities, such as the proportion of natural killer cells (which are a subgroup of lymphocytes that play an essential role in the cellular immune defense against virus-infected cells, bacteria, or tumor cells) among peripheral circulating lymphocytes [12,13]. The medial part of the prefrontal cortex, including the medial OFC (mOFC), vmPFC, and anterior cingulate cortex (ACC), has extensive outputs onto brain regions that regulate the secretion of peripheral hormones and cytokines via autonomic nervous functions, such as those of the hypothalamus, periaqueductal gray, amygdala, and thalamus [16,17]. Furthermore, previous studies have shown that connections between the olfactory bulb, primary olfactory cortex, and olfactory-related areas of the orbital cortex [18] and the mOFC may contribute to the hedonic experience associated with the processing of highly valued rewards [19], thereby suggesting that the mOFC represents the positive affective aspects of olfactory stimuli. Therefore, odor-evoked autobiographic memory accompanied by positive emotions might activate the mOFC and modulate peripheral circulating cytokine levels via the function of the mOFC; however, this phenomenon has not yet been demonstrated.

On the basis of these previous observations, in this study, we attempted to identify an association between brain activity and peripheral immune parameters (interleukin-2 [IL-2], interleukin-4 [IL-4], interleukin-6 [IL-6], interleukin-10 [IL-10], TNF-α, and IFN-γ levels) by simultaneously recording brain activity and plasma levels of cytokines by using positron emission tomography (PET) when the participants experienced an odor-evoked autobiographic memory.

## Materials and Methods

### Participants

The participants were recruited at the Nagoya University after the study had been approved by the local Ethics Committee (protocol number: 301). Because previous studies has suggested that the prevalence rate of olfactory evoked autobiographical memories is low (about 16%) [20,21], it was necessary to pre-select individuals for the PET study. Using the questionnaire, which asked the autobiographical odor episodes, we recruited participants who were able to retrieve clearly autobiographical odor episodes, which could be located with relatively exact space and time references. On the basis of their answers to the questionnaire, we selected 10 healthy volunteers (3 men and 7 women) for inclusion in this study. The age range of the participants was 20–35 years, and they all provided written informed consent in accordance with the Declaration of Helsinki. The participants received no medication during the experimental period. Women were examined during the late luteal and first follicular phases of their menstrual cycle, when the secretion of female sex hormones is low, thus minimizing the influence of these hormones on the endocrine and immune systems.

### Test Stimuli

A day before the experiment, the participants themselves selected the odor that evoked an autobiographic memory. These odors were referred to as nostalgic stimuli and included Givenchy Insense Ultramarine, Emanuel Ungaro Apparition Sky, Very Irresistible Givenchy Summer Cocktail, Angel Heart, Burberry Brit Eau De Parfum, and Angel Heart Lion Heart. The control stimuli were generic unmarketed perfumes (obtained from Kao Corporation) and were the same for all participants, as described previously [10]. We used 2 distinct odors as control stimuli; pretesting using 54 healthy volunteers established that these control odors did not evoke a sense of nostalgia nor an autobiographic memory.

### Experimental Procedures

Participants were instructed not to eat 2 h before the experiment, but they were allowed to consume nonalcoholic and caffeine-free fluids. Each participant lay down on the PET examining table, after which a heparinized catheter was inserted into his/her right forearm vein, for blood collection. A heparinized catheter that was used to administer the PET tracer was also inserted in the antecubital fossa vein in the left forearm. The participant was given instructions prior to the commencement of the experiment. After a 10-min transmission scan was completed, the experiment was started. A 4-min rest period was followed by the presentation of either the control odor (Control condition), the nostalgic odor that would evoke an autobiographic memory (Proust condition), or an air-only odor (Low-Level Control [LLC] condition) to the participant for 2 min by using an olfactometer consisting of 6 identical channels that were adjusted to deliver a constant flow rate of 1.3 L/min of odorized air from a diaphragm compressor (Aromageur; MIRAPRO Co., Ltd., Japan). A PET scan (duration, 60 s) was performed during this odor-smelling period. After the participant experienced the smell, his/her blood sample was collected, and the mood state and arousal level were assessed within 2 min. Assessments of test stimuli were also conducted. This was followed by an 11-min rest period, after which the next odor-smelling session started. These 3 conditions were presented twice to each participant. Therefore, all participants underwent 6 PET scans. The order of presentation of the 3 conditions was counterbalanced across participants.

### Assessments of Test Stimuli, Mood State, and Arousal Level

The perceived intensity of the test stimuli and evocations of a sense of nostalgia and autobiographic memory were evaluated by rating them on the visual analog scale (VAS), as follows. Intensity: completely scentless, 0% and extremely high intensity, 100%; nostalgia: no nostalgia, 0% and extremely high nostalgia, 100%; and memory: no memory evocation, 0% and extremely high memory evocation, 100%. Furthermore, to evaluate the mood state and arousal level of the participants, they were asked to evaluate subjectively their present pleasant mood and arousal level by rating it on the VAS, as follows. Mood state: extremely negative, 0%; neither positive nor negative, 50%; extremely positive, 100%; and arousal: extremely sleepy, 0%; neither sleepy nor aroused, 50%; extremely high arousal, 100%. In addition, we checked the content of the retrieved episodes cued by odors, and confirmed that the participants could retrieve autobiographic episodes, which could be located with relatively exact space and time references, by the nostalgic odors.

### Measurements of Cytokine Concentrations

The blood samples that were taken to measure plasma cytokine levels were anticoagulated with ethylenediaminetetraacetate, chilled, and centrifuged. The plasma was then removed and frozen at −80°C until analysis. Plasma cytokines (IL-2, IL-4, IL-6, IL-10, TNF-α, and IFN-γ) were determined using a BD cytometric bead array (Human Th1/Th2 Cytokine Kit II; BD Biosciences, San Diego, CA, USA) according to the manufacturer’s instructions. The intraassay coefficient of variation was less than 6%, and the interassay variation was less than 9% for the measurement of these cytokines.

### Image Acquisition by PET

During each block, the distribution of regional cerebral blood flow (rCBF) was measured on a General Electric, Advance NXi PET scanner (GE Healthcare Life Sciences, Little Chalfont, England) operated in a high-sensitivity three-dimensional mode. A venous catheter (for administering the tracer) was inserted into the antecubital fossa vein of the left forearm. After the subject’s head was positioned in the inflatable plastic head holder to prevent possible head movements, a 10-min transmission scan using a rotating ^68^Ge pin source was completed. In each block, after a 370-MBq bolus injection of H_2_^15^O over 30 s, scanning was started and continued for 60 s. Bolus injection was started 60 s after the initiation of the block. The integrated radioactivity accumulated during the 60 s of scanning was used as the index of rCBF. Six scans were acquired per subject, and the interval between successive scans was 15 min, to allow for radioactive levels to return to baseline. A Hanning filter was used to reconstruct images into 35 planes with a thickness of 4.5 mm and a resolution of 2 × 2 mm (full width at half maximum).

### Image Processing and Analysis

We used SPM8 revision 3684 (The Wellcome Trust Centre for Neuroimaging; http://www.fil.ion.ucl.ac.uk/spm) in MATLAB 2010a (MathWorks Inc., Natick, MA, USA) to analyze functional images. Images were initially realigned using sinc interpolation to remove artifacts before being transformed into a standard stereotactic space. Images were corrected for whole-brain global blood flow by proportional scaling and were smoothed using a Gaussian kernel to a final in-plane resolution of 8 mm at full width at half maximum. To identify significant regional changes in the Proust condition, differences between the 3 conditions (Proust, Control, and LLC) were analyzed by subtracting the images of the control and LLC conditions from the images of the Proust condition. The effects at each voxel were estimated using a general linear model. Voxel values for each contrast yielded a statistical parametric map of the t statistic (SPM t), which was subsequently transformed to a unit normal distribution (SPM z). The peak voxel value significance thresholds were set at p < 0.001 (uncorrected), and the cluster significance thresholds were set at p < 0.05 (uncorrected).

### Statistical Analyses of Psychological and Physiological Data

Results are expressed as the mean ± standard error of mean (SEM). The control and nostalgic stimuli were compared using paired *t* tests. Furthermore, Pearson correlation coefficients were computed between the beta values of significant brain regions and physiological indices, to examine the relationships among brain and physiological activities.

### Ethics Statement

This study was approved by the Ethics Committees of Nagoya University, Kizawa Memorial Hospital, and Kao Corporation. The participants of this study all provided written informed consent in accordance with the Declaration of Helsinki.

## Results

### Psychological Data

**Figure 1:**
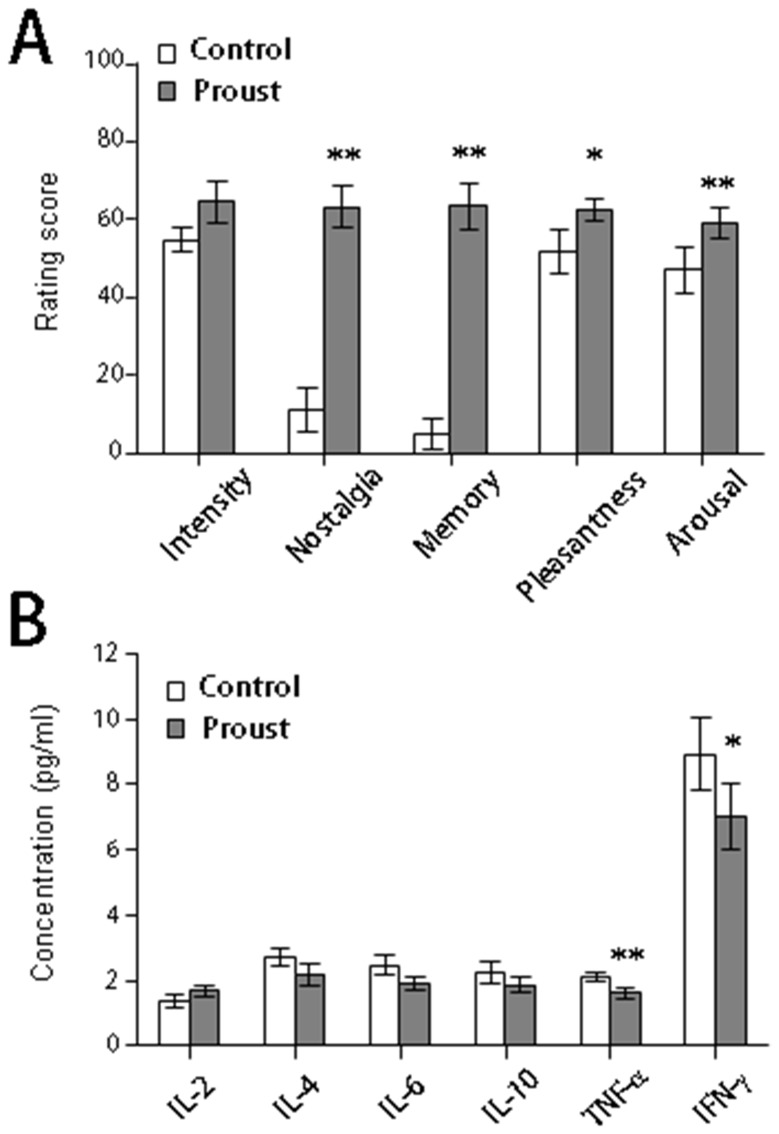
Psychological and plasma cytokine data. (A) Differences in the rating scores for the characteristics of control and nostalgic odors. **p < 0.01 and *p < 0.05 vs. control, paired *t* test. (B) Differences in the plasma concentration of cytokines (IL-2, IL-4, IL-6, IL-10, TNF-α, and IFN-γ) between control and nostalgic odors. **p < 0.01 and *p < 0.05 vs. control, paired *t* test.

To assess whether the test stimuli evoked an autobiographic memory, the participants were requested to evaluate the perceived intensity of test stimuli and evocations of sense of nostalgia and autobiographic memory by using the VAS. Although there were no significant differences in the rating score for perceived intensity, the scores for the sense of nostalgia (*df* = 9, *t* = -8.12, p < 0.01) and autobiographic memory (*df* = 9, *t* = -8.36, p < 0.01) were significantly greater in the Proust condition than in the control condition (Figure 1A). Mood states were also evaluated using the VAS. The paired *t* test revealed that the rating scores for mood state (pleasantness) (*df* = 9, *t* = -2.16, p < 0.05) and arousal level (*df* = 9, *t* = -2.50, p < 0.05) obtained after smelling the stimuli that induced the Proust condition were significantly higher than those obtained after smelling the control odor (Figure 1A).

### Effects of Odor-evoked Autobiographic Memory on Plasma Cytokines

To examine whether odor-evoked autobiographic memory influenced the levels of peripheral circulating cytokines, we measured the plasma concentrations of IL-2, IL-4, IL-6, IL-10, TNF-α, and IFN-γ after the smelling sessions (Figure 1B). The statistical analyses using paired *t* tests indicated that the plasma levels of TNF-α (*df* = 9, *t* = 3.52, p < 0.01) and IFN-γ (*df* = 9, *t* = 3.75, p < 0.01) were significantly lower after smelling the test stimulus that evoked the Proust condition than after the control condition (Figure 1B).

### Neuroimaging Data

**Figure 2:**
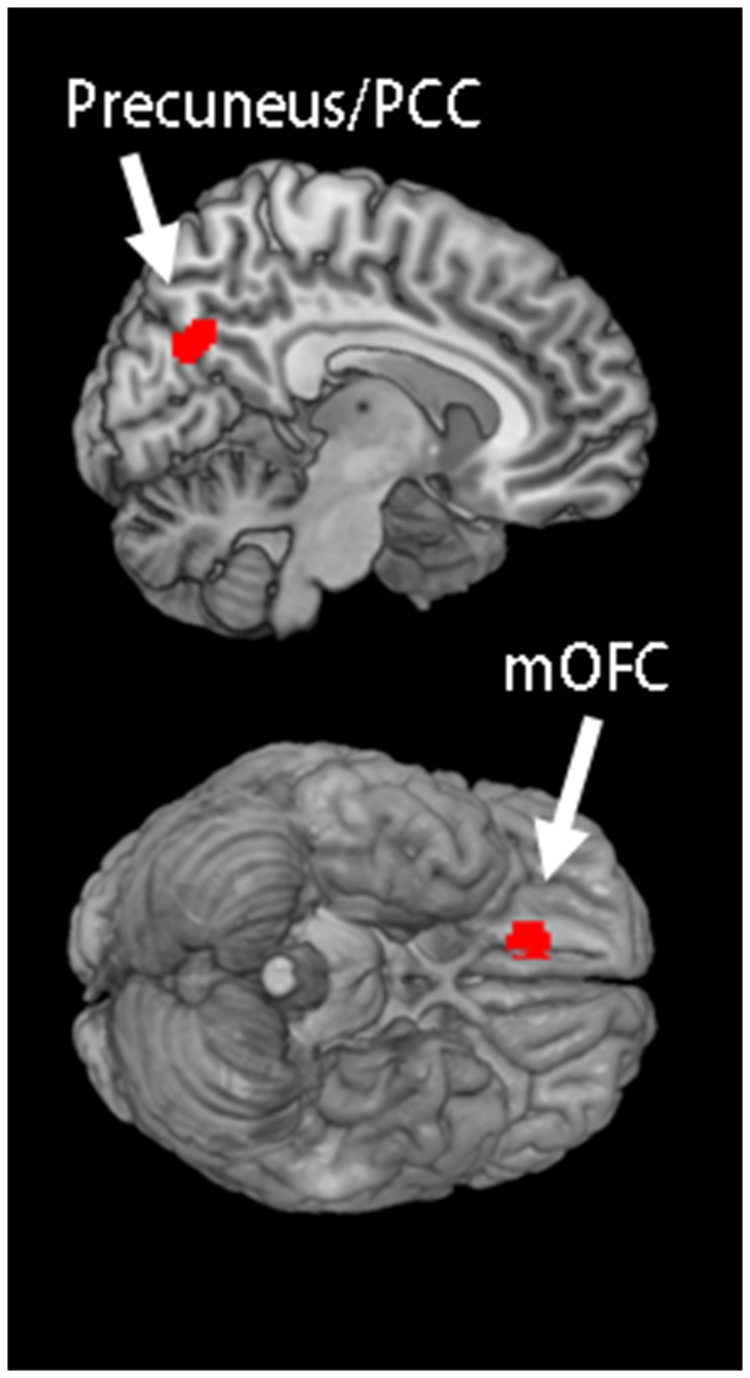
Neuroimaging analysis. Statistical parametric map showing a cluster that was activated significantly in the Proust condition compared with the control and LLC conditions. The statistical thresholds for the analysis were set at an uncorrected p < 0.001 at the voxel level, and at an uncorrected p < 0.05 at the cluster level.

**Figure 3:**
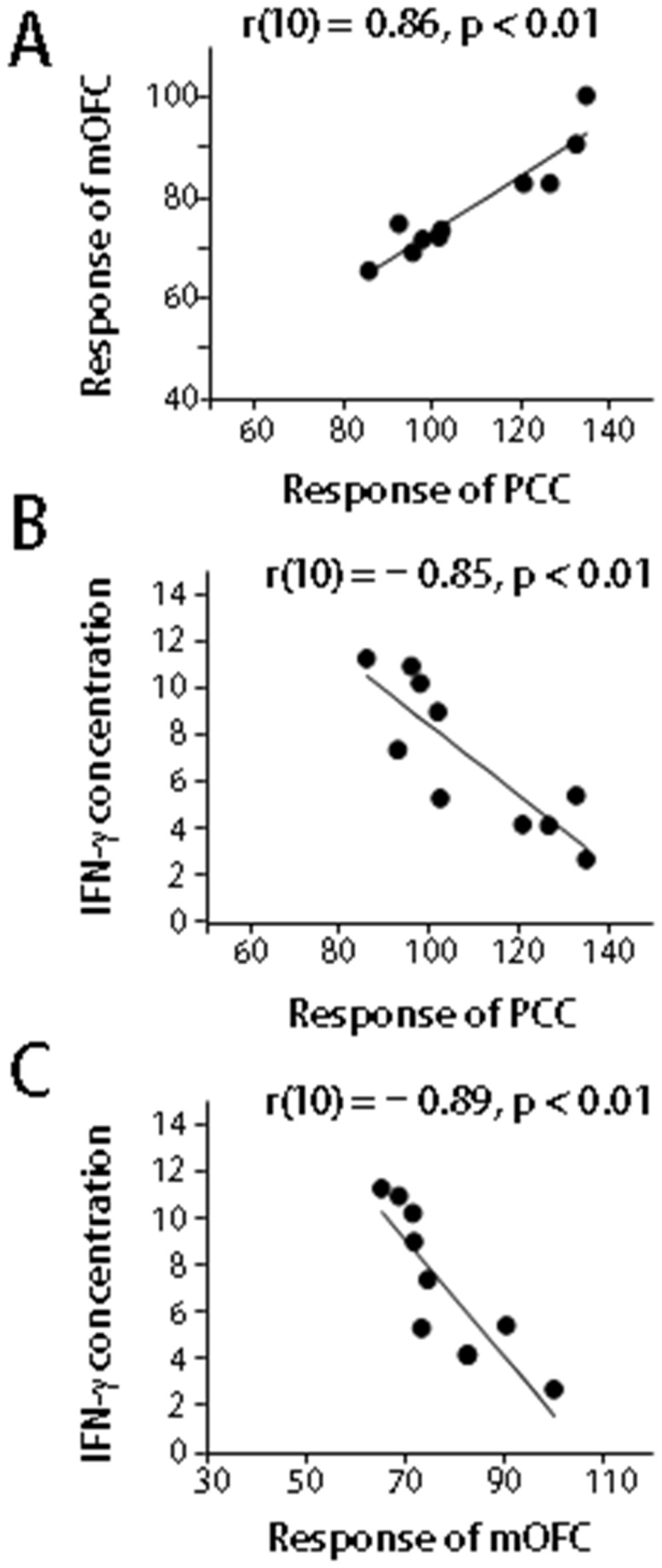
Correlation between peripheral cytokine levels and neuroimaging findings. (A) Scatter plot showing the correlation between the beta value of the mOFC cluster in the Proust condition and the beta value of the precuneus/PCC cluster in the Proust condition. (B) Scatter plot showing the correlation between the beta value of the precuneus/PCC cluster in the Proust condition and IFN-γ concentration after smelling a nostalgic odor that evoked an autobiographic memory. (C) Scatter plot showing the correlation between the beta value of the mOFC cluster in the Proust condition and IFN-γ concentration after smelling a nostalgic odor that evoked an autobiographic memory.

Subtraction of the control and LLC conditions from the Proust condition revealed significant increases in the rCBF of the mOFC (x, y, z = −12, 34, −26) and precuneus/PCC (x, y, z = 8, −68, 28) (Figure 2). We then analyzed the correlation between peripheral cytokine levels and rCBF. The correlation analyses using the beta values of the cluster within the mOFC and precuneus/PCC and plasma concentrations of TNF-α and IFN-γ indicated positive correlations between the mOFC response and the precuneus/PCC response (*r*(10) = 0.86, p < 0.01; Figure 3A). Furthermore, the plasma concentration of IFN-γ in the Proust condition was negatively correlated with the precuneus/PCC response (*r*(10) = −0.85, p < 0.01; Figure 3B) and with the mOFC response (*r*(10) = −0.89, p < 0.01; Figure 3C); however, no correlation between cytokine levels and responses of the precuneus/PCC and mOFC was observed in the control condition.

## Discussion

In the present study, we attempted to identify the neural underpinnings of the association between odor-evoked autobiographic memory accompanied by positive emotions and peripheral circulating cytokine levels by simultaneously recording brain activities and peripheral cytokine levels by using PET when the participants experienced an odor-evoked autobiographic memory. Participants experienced positive emotions and autobiographic memories when nostalgic odors were presented to them (Figure 1A). Peripheral proinflammatory cytokine levels, such as those of TNF-α and IFN-γ, were significantly reduced after the participants experienced odor-evoked autobiographic memories (Figure 1B). Subtraction analysis of PET images indicated that the mOFC and precuneus/PCC were significantly activated when the participants experienced odor-evoked autobiographic memories (Figure 2). Furthermore, a correlation analysis indicated the presence of a positive correlation between the activities of the mOFC and precuneus/PCC and IFN-γ concentration (Figure 3). Because the correlation between brain activity related to odor control stimuli and cytokines was not observed, the obtained relationship may not be driven by odor stimulation alone. These results suggest that the mOFC and precuneus/PCC play a role in regulating the secretion of peripheral proinflammatory cytokines when the participants experience an odor-evoked autobiographic memory accompanied by positive emotions.

Previous neuroimaging studies have indicated that the precuneus/PCC plays an important role in successful memory retrieval [22], and that odor-evoked autobiographic memory retrieval activates the precuneus/PCC [23]. Therefore, the precuneus/PCC activation shown in the present study might represent the retrieval of autobiographic memory. Furthermore, the mOFC represents the positive affective aspects of olfactory stimuli [18], and there is a neural connection between the PCC and mOFC [24]; therefore, the positive correlation between the PCC and mOFC shown in this study may represent the association between autobiographic memory retrieval and reward processing. Previous neuroimaging studies have shown that the mOFC regulates peripheral endocrine and immune functions [12,13]. These previous observations suggest that the neural networks involving the precuneus/PCC and mOFC might regulate the secretion of peripheral proinflammatory cytokines when a participant experiences odor-evoked autobiographic memory accompanied by positive emotions.

Herz and colleagues recently characterized odor-evoked autobiographic memories as being more emotional than those elicited by other sensory stimuli, as assessed using self-reporting and physiological responses [2–5]. Those authors used functional magnetic resonance imaging (fMRI) to demonstrate that the amygdala is strongly activated when autobiographic memories are evoked by olfactory cues than by other sensory cues, such as visual ones [5]. The amygdala is critical for the expression of emotion [6,7], and heightened activity of the amygdala represents an intense emotional experience. However, the present study did not show activation of the amygdala during the odor-evoked memory retrieval. The response speed of the amygdala may explain this absence of activation. The amygdala is quickly activated when an emotional stimulus is presented, and this activation then decreases quickly [25]. Because a relatively long scanning time is necessary for PET (60 s) acquisition than for fMRI, the quick response of the amygdala might not have been observed in the present study. In addition, our previous study indicated that the levels of the immunomodulatory cytokine IL-2 decreased after smelling a nostalgic odor compared with a control odor [10]. However, in this study, a decrease in IL-2 levels was not observed in the Proust condition; instead, we showed a decrease in the levels of proinflammatory cytokines, such as TNF-α and IFN-γ, in the Proust condition. What is the explanation for the difference observed between the present and previous studies? The PET conditions may be stressful compared with normal experimental conditions; therefore, it is possible that baseline systemic inflammation was increased by stress. IL-2, TNF-α, and IFN-γ are involved in immune-system signaling and systemic inflammation; however, recent studies have demonstrated that IL-2 is also essential for the downregulation of immune responses via the enhancement of anti-inflammatory cytokine secretion [26]. Thus, it is possible that IL-2 is actively produced in the stressful PET conditions to reduce systemic inflammation, and this would explain why we did not observe a difference in IL-2 concentration between the control and Proust conditions.

The mediators of the association between the neural network that includes the mOFC and precuneus/PCC and proinflammatory cytokines remain unclear. Previous studies have indicated that the activation of the cannabinoid receptor 2 (CB2) by a CB2-selective agonist seems to have anti-inflammatory properties via TNF-α and IFN-γ inhibition [27,28]. Furthermore, a previous study indicated that the endocannabinoid system is associated with positive emotion evocation [29,30]. Thus, stimulation of the endocannabinoid system by odor-evoked positive emotions may have suppressed proinflammatory cytokine secretion. To test this hypothesis, we will have to measure changes in peripheral endocannabinoid levels after a participant experiences the odor-evoked autobiographic memory accompanied by positive emotions in a future study.

This study has certain limitations. First, the relatively short experimental time (odor-smelling session, 2 min) was insufficient to determine the effects of the olfactory stimulation on the endocrine and immune systems, even though previous studies have reported significant changes in endocrine and immune parameters over a short time [8,11,31]. In previous studies, changes in peripheral cytokine levels were observed at least 30 min after the presentation of experimental stimuli; thus, the changes in peripheral cytokine levels shown in this study may have been too fast. However, previous studies have shown that peripheral circulating lymphocytes that secrete several cytokines can be rapidly redistributed after emotional arousal (about 1–2 min). Thus, the distribution of lymphocytes that secrete proinflammatory cytokines in the blood may be possibly changed after autobiographic memory evocation. The generalizability of the present findings must be tested further by using a longer experimental time and lymphocyte-distribution analyses. Second, some reports have noted sex differences regarding physiological reactivity [32]; however, we did not investigate interaction effects with sex. In addition, interaction effects with the order in which the 2 types of odors were presented may exist. In a future study, we will attempt to investigate the interaction effects with stimulus-presentation order and sex. Third, the obtained relationships between cytokine levels and brain activities may be driven by pleasantness rather than by retrieval of autobiographical odor information, because our previous study has indicated the relationship between positive emotions and cytokine levels [9]. Further, the nostalgic odor, rather than the control odors, might be familiar to the participants. A previous study has indicated that novel odors affect the gene expression of cytokines in the rat brain [33]. Thus, odor familiarity may have had confounding effects on the obtained findings. In a future study, we will attempt to investigate the effects of pleasantness and familiarity on the obtained relationships between cytokine levels and brain activities by using a full factorial design.

The results of the present study suggest the presence of a brain–immune interaction during the experience of an odor-evoked autobiographic memory accompanied by positive emotions. The neural networks that include the mOFC and precuneus/PCC might play a key role in regulating the secretion of peripheral proinflammatory cytokines during the experience of an odor-evoked autobiographic memory accompanied by positive emotions. Odor-evoked autobiographic memories may have beneficial effects on human health and well-being via the inhibition of systemic inflammation.
